# (1*H*-1,3-Benzimidazole-5,6-dicarboxylic acid)(5-carboxyl­ato-1*H*-1,3-benzimidazole-6-carboxylic acid)silver(I) monohydrate

**DOI:** 10.1107/S1600536809044535

**Published:** 2009-10-31

**Authors:** Hong Zhai

**Affiliations:** a College of Chemistry & Chemical Engineering, Shanxi Datong University, Shanxi 037009, People’s Republic of China

## Abstract

The title compound, [Ag(C_9_H_5_N_2_O_4_)(C_9_H_6_N_2_O_4_)]·H_2_O, contains one independent Ag atom, a neutral 1*H*-benzimidazole-5,6-dicarboxylic acid (bdcH), its monodeprotonated form, *i.e.* 5-carboxyl­ato-1*H*-1,3-benzimidazole-6-carboxylic acid (bdc), and one solvent water mol­ecule, the latter being disordered over three sites with site occupancy factors of 0.375 (× 2) and 0.25. In addition, the H atom on one carboxylic acid residue is disordered, being connected to each of the O atoms 50% of the time. The Ag atom is in a virtually linear geometry defined by two N atoms derived from the bdc and bdcH ligands. The three-dimensional supra­molecular structure is stablized by extensive O—H⋯O and N—H⋯O hydrogen bonds. An intramolecular O—H⋯O hydrogen bond is also present.

## Related literature

For related structures, see: Gao *et al.* (2008 ▶); Li *et al.* (2009 ▶); Lo *et al.* (2007 ▶); Wei *et al.* (2008 ▶); Yao *et al.* (2008 ▶).
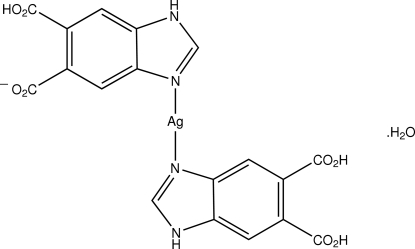


## Experimental

### 

#### Crystal data

[Ag(C_9_H_5_N_2_O_4_)(C_9_H_6_N_4_O_2_)]·H_2_O
*M* *_r_* = 537.37
Monoclinic, 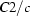

*a* = 28.483 (3) Å
*b* = 18.6398 (17) Å
*c* = 7.2251 (7) Å
β = 99.046 (1)°
*V* = 3788.2 (6) Å^3^
*Z* = 8
Mo *K*α radiation
μ = 1.13 mm^−1^
*T* = 298 K
0.31 × 0.23 × 0.19 mm

#### Data collection

Bruker APEXII area-detector diffractometer
Absorption correction: multi-scan (*SADABS*; Sheldrick, 2004 ▶) *T*_min_ = 0.740, *T*_max_ = 0.807
10329 measured reflections
3675 independent reflections
2572 reflections with *I* > 2σ(*I*)
*R*_int_ = 0.044

#### Refinement

*R*[*F* ^2^ > 2σ(*F* ^2^)] = 0.039
*wR*(*F* ^2^) = 0.092
*S* = 1.04
3675 reflections
307 parameters
18 restraints
H-atom parameters constrained
Δρ_max_ = 0.52 e Å^−3^
Δρ_min_ = −0.59 e Å^−3^

### 

Data collection: *APEX2* (Bruker, 2004 ▶); cell refinement: *SAINT* (Bruker, 2004 ▶); data reduction: *SAINT*; program(s) used to solve structure: *SHELXS97* (Sheldrick, 2008 ▶); program(s) used to refine structure: *SHELXL97* (Sheldrick, 2008 ▶); molecular graphics: *XP* in *SHELXTL* (Sheldrick, 2008 ▶); software used to prepare material for publication: *SHELXL97*.

## Supplementary Material

Crystal structure: contains datablocks I, global. DOI: 10.1107/S1600536809044535/tk2558sup1.cif

Structure factors: contains datablocks I. DOI: 10.1107/S1600536809044535/tk2558Isup2.hkl

Additional supplementary materials:  crystallographic information; 3D view; checkCIF report

## Figures and Tables

**Table 1:** Hydrogen-bond geometry (Å, °)

*D*—H⋯*A*	*D*—H	H⋯*A*	*D*⋯*A*	*D*—H⋯*A*
O1—H1⋯O7^i^	0.85	1.77	2.603 (4)	168
O3—H3⋯O3^ii^	0.85	1.71	2.528 (5)	162
O4—H4⋯O4^iii^	0.85	1.66	2.500 (6)	168
O7—H7⋯O5	0.85	1.54	2.389 (4)	176
N2—H2*A*⋯O6^iv^	0.86	1.88	2.733 (4)	173
N4—H4*A*⋯O8^v^	0.86	2.04	2.805 (4)	148

Symmetry codes: (i) 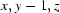; (ii) 
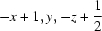
; (iii) 
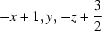
; (iv) 
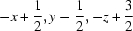
; (v) 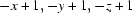.

**Fig. 1.**
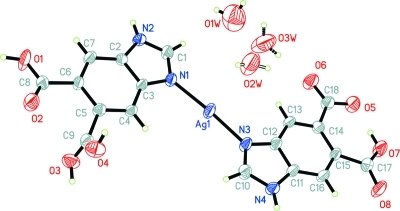
Displacement ellipsoid plot (50% probability level) of (I), with atom numbering. The water molecule is fractionally occupied with site occupancy factors of 0.375, 0.375 and 0.25.

**Fig. 2.**
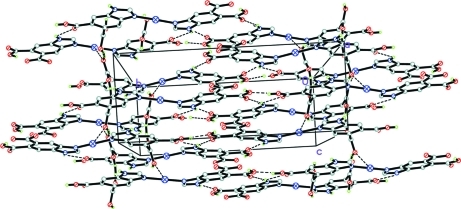
The packing diagram of (I), with partially-occupied H atoms omitted for clarity. Hydrogen bonds are shown as dashed lines.

## supplementary crystallographic information

### Comment

N-Heterocyclic carboxylic acids as organic ligands attract attention not only because of versatile coordination modes but also owing to its ability to facilitate the formation of high-dimensional coordination polymers. One such example, namely, 1*H*-benzimidazole-5,6-dicarboxylic acid (bdcH), is a semi-rigid, multidentate ligand that can provide up to six donor atoms (two N and four O atoms) with variable coordination modes. This is therefore considered as an excellent candidate for generating 3-D architectures. Up to now, the reported complexes based on the bdc ligand are rare but have attracted recent interest (Lo *et al.*, 2007; Gao *et al.*, 2008; Wei *et al.*, 2008; Yao *et al.*, 2008; Li *et al.*, 2009). Herein, the first Ag supramolecular compound based on the bdc ligand, namely [Ag(C_9_H_5_N_2_O_2_)(C_9_H_6_N_2_O_2_)].H_2_O, (I), is reported.

As is shown in Fig. 1, the asymmetric unit consists of bdcH and bdc ligands, one Ag atom, and one solvent water molecule. The water molecule is disordered over three sites with site occupancy factors = 0.375 (x 2) and 0.25, see Experimental. The Ag atom has a linear coordination environment being bound to two N atoms derived from the bdc ligands.

A packing diagram showing the 3-D supramolecular structure arising from a larg e number of hydrogen bonding interactions is shown in Fig. 2. Through the agency of intermolecular hydrogen bond interactions involving the bdc and bdcH ligands, Table 1, a layer structure is generated. These are connected into a 3-D network via hydrogen bonding interactions involving the water molecules.

### Experimental

A mixture of the bdc (0.0415 g, 0.20 mmol), AgNO_3_ (0.0340 g, 0.20 mmol) and water (10 ml) was heated to 430 K for 72 h in a 23 ml Teflon-lined stainless-steel autoclave. After the reaction, the bomb was cooled to room temperature in a rate of 278 K per hour. Colourless prismatic crystals were collected and dried in air.

### Refinement

For the bdc ligand, all H atoms were placed at calculated positions and were treated as riding on the parent atoms with C—H = 0.93 and O—H = 0.86 Å, and with *U*_iso_(H) = 1.2 or 1.5 *U*_eq_(C, O). The H atom on the carboxylic acid residue with the O3 and O4 atoms was disordered. This was modelled over two sites of equal weight.

The solvent water molecule was also disordered over three positions, with site occupancy factors of 0.375, 0.375 and 0.25, respectively. The H atoms were included for each partially occupied molecule with O—H distances of 0.85 Å, and with *U*ĩso~(H) = 1.2*U*~eq~(O).

### Crystal data

**Table:**

[Ag(C_9_H_5_N_2_O_4_)(C_9_H_6_N_2_O_4_)]·H_2_O	*F*(000) = 2145.0
*M**_r_* = 537.37	*D*_x_ = 1.885 Mg m^−^^3^
Monoclinic, *C*2/*c*	Mo *K*α radiation, λ = 0.71073 Å
Hall symbol: -C 2yc	Cell parameters from 2071 reflections
*a* = 28.483 (3) Å	θ = 2.4–22.4°
*b* = 18.6398 (17) Å	µ = 1.13 mm^−^^1^
*c* = 7.2251 (7) Å	*T* = 298 K
β = 99.046 (1)°	Block, colourless
*V* = 3788.2 (6) Å^3^	0.31 × 0.23 × 0.19 mm
*Z* = 8	

### Data collection

**Table:**

Bruker APEXII area-detector diffractometer	3675 independent reflections
Radiation source: fine-focus sealed tube	2572 reflections with *I* > 2σ(*I*)
graphite	*R*_int_ = 0.044
φ and ω scans	θ_max_ = 26.0°, θ_min_ = 2.4°
Absorption correction: multi-scan (*SADABS*; Sheldrick, 2004)	*h* = −35→35
*T*_min_ = 0.740, *T*_max_ = 0.807	*k* = −20→22
10329 measured reflections	*l* = −8→7

### Refinement

**Table:**

Refinement on *F*^2^	Primary atom site location: structure-invariant direct methods
Least-squares matrix: full	Secondary atom site location: difference Fourier map
*R*[*F*^2^ > 2σ(*F*^2^)] = 0.039	Hydrogen site location: inferred from neighbouring sites
*wR*(*F*^2^) = 0.092	H-atom parameters constrained
*S* = 1.04	*w* = 1/[σ^2^(*F*_o_^2^) + (0.04*P*)^2^] where *P* = (*F*_o_^2^ + 2*F*_c_^2^)/3
3675 reflections	(Δ/σ)_max_ = 0.001
307 parameters	Δρ_max_ = 0.52 e Å^−^^3^
18 restraints	Δρ_min_ = −0.59 e Å^−^^3^

### Special details

**Table:**

Geometry. All e.s.d.'s (except the e.s.d. in the dihedral angle between two l.s. planes) are estimated using the full covariance matrix. The cell e.s.d.'s are taken into account individually in the estimation of e.s.d.'s in distances, angles and torsion angles; correlations between e.s.d.'s in cell parameters are only used when they are defined by crystal symmetry. An approximate (isotropic) treatment of cell e.s.d.'s is used for estimating e.s.d.'s involving l.s. planes.
Refinement. Refinement of *F*^2^ against ALL reflections. The weighted *R*-factor *wR* and goodness of fit *S* are based on *F*^2^, conventional *R*-factors *R* are based on *F*, with *F* set to zero for negative *F*^2^. The threshold expression of *F*^2^ > σ(*F*^2^) is used only for calculating *R*-factors(gt) *etc*. and is not relevant to the choice of reflections for refinement. *R*-factors based on *F*^2^ are statistically about twice as large as those based on *F*, and *R*- factors based on ALL data will be even larger.

### Fractional atomic coordinates and isotropic or equivalent isotropic displacement parameters (Å^2^)

**Table:**

	*x*	*y*	*z*	*U*_iso_*/*U*_eq_	Occ. (<1)
Ag1	0.374338 (11)	0.168833 (16)	0.58373 (5)	0.04290 (14)	
O1	0.36561 (10)	−0.26003 (15)	0.5821 (5)	0.0606 (10)	
H1	0.3710	−0.3049	0.5832	0.073*	
O2	0.44076 (10)	−0.23159 (15)	0.5698 (5)	0.0547 (9)	
O3	0.45996 (10)	−0.11508 (16)	0.3045 (4)	0.0470 (8)	
H3	0.4863	−0.1056	0.2688	0.056*	0.50
O4	0.50175 (10)	−0.08915 (16)	0.5782 (4)	0.0491 (8)	
H4	0.5006	−0.0830	0.6940	0.059*	0.50
O5	0.30757 (10)	0.53188 (16)	0.6824 (5)	0.0548 (9)	
O6	0.28554 (11)	0.42025 (16)	0.6978 (5)	0.0580 (10)	
O7	0.37366 (11)	0.60136 (15)	0.6187 (5)	0.0538 (9)	
H7	0.3493	0.5781	0.6386	0.065*	
O8	0.44651 (11)	0.58811 (16)	0.5706 (5)	0.0612 (10)	
N1	0.33935 (11)	0.07120 (16)	0.6181 (5)	0.0314 (8)	
N2	0.28969 (11)	−0.01398 (17)	0.6799 (5)	0.0338 (8)	
H2A	0.2646	−0.0338	0.7095	0.041*	
N3	0.41656 (11)	0.25914 (16)	0.5533 (5)	0.0384 (9)	
N4	0.47918 (11)	0.32435 (17)	0.5099 (5)	0.0395 (9)	
H4A	0.5071	0.3347	0.4871	0.047*	
C1	0.29744 (13)	0.0560 (2)	0.6672 (6)	0.0354 (10)	
H1A	0.2757	0.0908	0.6905	0.043*	
C2	0.32928 (12)	−0.0490 (2)	0.6369 (6)	0.0287 (9)	
C3	0.36007 (13)	0.00500 (19)	0.5978 (6)	0.0275 (9)	
C4	0.40427 (13)	−0.01206 (19)	0.5503 (6)	0.0288 (9)	
H4B	0.4251	0.0237	0.5247	0.035*	
C5	0.41601 (13)	−0.0831 (2)	0.5424 (6)	0.0301 (9)	
C6	0.38460 (13)	−0.1378 (2)	0.5819 (6)	0.0293 (9)	
C7	0.34103 (14)	−0.1213 (2)	0.6295 (6)	0.0349 (10)	
H7A	0.3202	−0.1569	0.6557	0.042*	
C8	0.40022 (15)	−0.2145 (2)	0.5758 (6)	0.0366 (10)	
C9	0.46214 (15)	−0.0985 (2)	0.4738 (7)	0.0392 (11)	
C10	0.46025 (15)	0.2587 (2)	0.5131 (7)	0.0428 (11)	
H10A	0.4762	0.2170	0.4895	0.051*	
C11	0.44556 (13)	0.3720 (2)	0.5500 (6)	0.0327 (10)	
C12	0.40613 (13)	0.3312 (2)	0.5766 (6)	0.0327 (9)	
C13	0.36540 (13)	0.3643 (2)	0.6177 (6)	0.0327 (10)	
H13A	0.3396	0.3367	0.6393	0.039*	
C14	0.36314 (13)	0.4380 (2)	0.6266 (6)	0.0321 (10)	
C15	0.40400 (14)	0.47992 (19)	0.5986 (6)	0.0314 (9)	
C16	0.44451 (14)	0.4463 (2)	0.5622 (6)	0.0352 (10)	
H16A	0.4711	0.4732	0.5458	0.042*	
C17	0.40890 (16)	0.5620 (2)	0.5952 (6)	0.0395 (11)	
C18	0.31614 (14)	0.4648 (2)	0.6704 (6)	0.0399 (11)	
O1W	0.2366 (4)	0.1989 (7)	0.851 (2)	0.126 (5)	0.38
H1C	0.2379	0.2042	0.9681	0.151*	0.38
H1D	0.2255	0.1565	0.8423	0.151*	0.38
O2W	0.2352 (4)	0.2876 (6)	0.5630 (19)	0.108 (4)	0.38
H2C	0.2495	0.3257	0.6052	0.129*	0.38
H2D	0.2530	0.2509	0.5812	0.129*	0.38
O3W	0.2637 (6)	0.2800 (10)	0.809 (3)	0.110 (6)	0.25
H3C	0.2702	0.3223	0.7767	0.131*	0.25
H3D	0.2564	0.2647	0.9111	0.131*	0.25

### Atomic displacement parameters (Å^2^)

**Table:**

	*U*^11^	*U*^22^	*U*^33^	*U*^12^	*U*^13^	*U*^23^
Ag1	0.0433 (2)	0.02077 (18)	0.0675 (3)	−0.00497 (15)	0.01755 (17)	0.00033 (16)
O1	0.0401 (18)	0.0188 (16)	0.126 (3)	−0.0020 (13)	0.0233 (19)	0.0014 (17)
O2	0.0414 (18)	0.0328 (18)	0.096 (3)	0.0092 (14)	0.0283 (17)	0.0062 (17)
O3	0.0345 (16)	0.059 (2)	0.051 (2)	−0.0025 (14)	0.0147 (15)	−0.0074 (16)
O4	0.0297 (16)	0.064 (2)	0.054 (2)	−0.0008 (15)	0.0076 (15)	0.0002 (16)
O5	0.0422 (18)	0.0341 (19)	0.093 (3)	0.0100 (14)	0.0259 (18)	−0.0047 (17)
O6	0.0364 (17)	0.0408 (19)	0.104 (3)	−0.0013 (15)	0.0317 (18)	−0.0021 (18)
O7	0.0478 (19)	0.0226 (16)	0.096 (3)	0.0029 (14)	0.0256 (19)	0.0006 (16)
O8	0.049 (2)	0.0314 (19)	0.110 (3)	−0.0119 (15)	0.032 (2)	−0.0032 (18)
N1	0.0261 (18)	0.0219 (17)	0.047 (2)	−0.0002 (14)	0.0087 (15)	−0.0005 (15)
N2	0.0201 (16)	0.033 (2)	0.051 (2)	−0.0036 (14)	0.0140 (15)	−0.0005 (16)
N3	0.0298 (18)	0.0201 (18)	0.068 (3)	0.0007 (14)	0.0167 (17)	0.0010 (16)
N4	0.0279 (18)	0.030 (2)	0.066 (3)	0.0014 (15)	0.0215 (17)	0.0003 (17)
C1	0.028 (2)	0.028 (2)	0.051 (3)	0.0021 (18)	0.008 (2)	−0.0022 (19)
C2	0.0202 (19)	0.028 (2)	0.039 (3)	−0.0009 (16)	0.0099 (17)	−0.0025 (18)
C3	0.0240 (19)	0.022 (2)	0.037 (2)	−0.0045 (16)	0.0071 (17)	0.0005 (17)
C4	0.0220 (19)	0.024 (2)	0.042 (3)	−0.0034 (16)	0.0110 (18)	0.0015 (17)
C5	0.024 (2)	0.030 (2)	0.037 (3)	−0.0024 (17)	0.0052 (17)	0.0002 (18)
C6	0.031 (2)	0.0189 (19)	0.038 (3)	−0.0007 (16)	0.0056 (19)	0.0010 (17)
C7	0.032 (2)	0.026 (2)	0.049 (3)	−0.0042 (18)	0.012 (2)	0.0023 (19)
C8	0.035 (2)	0.027 (2)	0.052 (3)	0.0037 (19)	0.017 (2)	−0.0011 (19)
C9	0.034 (2)	0.029 (2)	0.057 (3)	0.0048 (19)	0.013 (2)	−0.001 (2)
C10	0.038 (2)	0.026 (2)	0.068 (3)	0.0064 (19)	0.018 (2)	−0.001 (2)
C11	0.028 (2)	0.025 (2)	0.047 (3)	−0.0020 (17)	0.0123 (19)	0.0020 (18)
C12	0.033 (2)	0.022 (2)	0.045 (3)	0.0006 (18)	0.0105 (18)	0.0026 (19)
C13	0.028 (2)	0.024 (2)	0.049 (3)	−0.0010 (17)	0.0154 (19)	0.0035 (18)
C14	0.028 (2)	0.028 (2)	0.042 (3)	0.0061 (17)	0.0098 (18)	−0.0003 (18)
C15	0.033 (2)	0.021 (2)	0.042 (3)	−0.0025 (17)	0.0095 (19)	0.0004 (17)
C16	0.029 (2)	0.026 (2)	0.052 (3)	−0.0052 (17)	0.013 (2)	0.0035 (19)
C17	0.045 (3)	0.029 (2)	0.045 (3)	−0.003 (2)	0.011 (2)	−0.001 (2)
C18	0.031 (2)	0.035 (3)	0.055 (3)	0.001 (2)	0.011 (2)	0.000 (2)
O1W	0.100 (7)	0.111 (7)	0.168 (9)	0.017 (6)	0.025 (7)	−0.013 (7)
O2W	0.096 (7)	0.071 (6)	0.158 (9)	−0.017 (6)	0.025 (6)	0.000 (6)
O3W	0.095 (9)	0.102 (9)	0.142 (10)	−0.019 (7)	0.050 (8)	0.029 (8)

### Geometric parameters (Å, °)

**Table:**

Ag1—N3	2.100 (3)	C2—C3	1.393 (5)
Ag1—N1	2.108 (3)	C3—C4	1.393 (5)
O1—C8	1.307 (5)	C4—C5	1.369 (5)
O1—H1	0.8498	C4—H4B	0.9300
O2—C8	1.205 (5)	C5—C6	1.415 (5)
O3—C9	1.254 (5)	C5—C9	1.503 (5)
O3—H3	0.8499	C6—C7	1.374 (5)
O4—C9	1.267 (5)	C6—C8	1.500 (5)
O4—H4	0.8500	C7—H7A	0.9300
O5—C18	1.280 (5)	C10—H10A	0.9300
O6—C18	1.242 (5)	C11—C16	1.388 (5)
O7—C17	1.277 (5)	C11—C12	1.394 (5)
O7—H7	0.8499	C12—C13	1.387 (5)
O8—C17	1.215 (5)	C13—C14	1.378 (5)
N1—C1	1.329 (5)	C13—H13A	0.9300
N1—C3	1.385 (5)	C14—C15	1.442 (5)
N2—C1	1.328 (5)	C14—C18	1.508 (5)
N2—C2	1.380 (4)	C15—C16	1.375 (5)
N2—H2A	0.8600	C15—C17	1.536 (5)
N3—C10	1.322 (5)	C16—H16A	0.9300
N3—C12	1.391 (5)	O1W—H1C	0.8500
N4—C10	1.339 (5)	O1W—H1D	0.8501
N4—C11	1.370 (5)	O2W—H2C	0.8501
N4—H4A	0.8600	O2W—H2D	0.8501
C1—H1A	0.9300	O3W—H3C	0.8496
C2—C7	1.391 (5)	O3W—H3D	0.8496
			
N3—Ag1—N1	173.32 (12)	C2—C7—H7A	121.3
C8—O1—H1	120.1	O2—C8—O1	124.2 (4)
C9—O3—H3	109.5	O2—C8—C6	122.9 (4)
C9—O4—H4	115.8	O1—C8—C6	112.9 (3)
C17—O7—H7	114.2	O3—C9—O4	121.2 (4)
C1—N1—C3	104.7 (3)	O3—C9—C5	117.2 (4)
C1—N1—Ag1	132.6 (3)	O4—C9—C5	121.4 (4)
C3—N1—Ag1	122.6 (2)	N3—C10—N4	113.2 (3)
C1—N2—C2	107.4 (3)	N3—C10—H10A	123.4
C1—N2—H2A	126.3	N4—C10—H10A	123.4
C2—N2—H2A	126.3	N4—C11—C16	133.1 (4)
C10—N3—C12	105.0 (3)	N4—C11—C12	106.3 (3)
C10—N3—Ag1	126.3 (3)	C16—C11—C12	120.6 (3)
C12—N3—Ag1	128.6 (2)	C13—C12—N3	131.1 (3)
C10—N4—C11	107.0 (3)	C13—C12—C11	120.4 (4)
C10—N4—H4A	126.5	N3—C12—C11	108.5 (3)
C11—N4—H4A	126.5	C14—C13—C12	120.0 (4)
N2—C1—N1	113.2 (3)	C14—C13—H13A	120.0
N2—C1—H1A	123.4	C12—C13—H13A	120.0
N1—C1—H1A	123.4	C13—C14—C15	119.3 (3)
N2—C2—C7	132.6 (3)	C13—C14—C18	112.9 (3)
N2—C2—C3	105.5 (3)	C15—C14—C18	127.8 (4)
C7—C2—C3	121.9 (3)	C16—C15—C14	120.0 (4)
N1—C3—C2	109.3 (3)	C16—C15—C17	111.7 (3)
N1—C3—C4	130.2 (3)	C14—C15—C17	128.2 (3)
C2—C3—C4	120.5 (3)	C15—C16—C11	119.6 (3)
C5—C4—C3	117.8 (3)	C15—C16—H16A	120.2
C5—C4—H4B	121.1	C11—C16—H16A	120.2
C3—C4—H4B	121.1	O8—C17—O7	121.2 (4)
C4—C5—C6	121.5 (3)	O8—C17—C15	119.1 (4)
C4—C5—C9	115.5 (3)	O7—C17—C15	119.7 (4)
C6—C5—C9	122.9 (3)	O6—C18—O5	119.8 (4)
C7—C6—C5	120.9 (3)	O6—C18—C14	118.7 (4)
C7—C6—C8	120.3 (3)	O5—C18—C14	121.5 (4)
C5—C6—C8	118.8 (3)	H1C—O1W—H1D	97.8
C6—C7—C2	117.3 (3)	H2C—O2W—H2D	112.1
C6—C7—H7A	121.3	H3C—O3W—H3D	130.0

### Hydrogen-bond geometry (Å, °)

**Table:**

*D*—H···*A*	*D*—H	H···*A*	*D*···*A*	*D*—H···*A*
O1—H1···O7^i^	0.85	1.77	2.603 (4)	168
O3—H3···O3^ii^	0.85	1.71	2.528 (5)	162
O4—H4···O4^iii^	0.85	1.66	2.500 (6)	168
O7—H7···O5	0.85	1.54	2.389 (4)	176
N2—H2A···O6^iv^	0.86	1.88	2.733 (4)	173
N4—H4A···O8^v^	0.86	2.04	2.805 (4)	148

Symmetry codes: (i) *x*, *y*−1, *z*; (ii) −*x*+1, *y*, −*z*+1/2; (iii) −*x*+1, *y*, −*z*+3/2; (iv) −*x*+1/2, *y*−1/2, −*z*+3/2; (v) −*x*+1, −*y*+1, −*z*+1.
